# Contributions of IFN-γ and granulysin to the clearance of *Plasmodium yoelii* blood stage

**DOI:** 10.1371/journal.ppat.1008840

**Published:** 2020-09-10

**Authors:** Natália Satchiko Hojo-Souza, Patrick Orestes de Azevedo, Júlia Teixeira de Castro, Andréa Teixeira-Carvalho, Judy Lieberman, Caroline Junqueira, Ricardo Tostes Gazzinelli

**Affiliations:** 1 Laboratório de Imunopatologia, Instituto René Rachou, Fundação Oswaldo Cruz, Belo Horizonte, MG, Brazil; 2 Departamento de Bioquímica e Imunologia, Universidade Federal de Minas Gerais, Belo Horizonte, MG, Brazil; 3 Grupo Integrado de Pesquisas em Biomarcadores, Instituto René Rachou, Fundação Oswaldo Cruz, Belo Horizonte, MG, Brazil; 4 Program in Cellular and Molecular Medicine, Boston Children’s Hospital and Department of Pediatrics, Harvard Medical School, Boston, MA, United States of America; 5 Division of Infectious Disease and Immunology, University of Massachusetts Medical School, Worcester, MA, United States of America; 6 Plataforma de Medicina Translacional, Fundação Oswaldo Cruz, Ribeirão Preto, SP, Brazil; National Institutes of Health, UNITED STATES

## Abstract

*P*. *vivax*-infected Retics (iRetics) express human leukocyte antigen class I (HLA-I), are recognized by CD8^+^ T cells and killed by granulysin (GNLY) and granzymes. However, how *Plasmodium* infection induces MHC-I expression on Retics is unknown. In addition, whether GNLY helps control *Plasmodium* infection *in vivo* has not been studied. Here, we examine these questions using rodent infection with the *P*. *yoelii* 17XNL strain, which has tropism for Retics. Infection with *P*. *yoelii* caused extramedullary erythropoiesis, reticulocytosis and expansion of CD8^+^CD44^+^CD62L^-^ IFN-γ-producing T cells that form immune synapses with iRetics. We now provide evidence that MHC-I expression by iRetic is dependent on IFN-γ-induced transcription of IRF-1, MHC-I and β2-microglobulin (β2-m) in erythroblasts. Consistently, CTLs from infected wild type (WT) mice formed immune synapses with iRetics in an IFN-γ- and MHC-I-dependent manner. When challenged with *P*. *yoelii* 17XNL, WT mice cleared parasitemia and survived, while IFN-γ KO mice remained parasitemic and all died. β2-m KO mice that do not express MHC-I and have virtually no CD8^+^ T cells had prolonged parasitemia, and 80% survived. Because mice do not express GNLY, *GNLY*-transgenic mice can be used to assess the *in vivo* importance of GNLY. Parasite clearance was accelerated in *GNLY*-transgenic mice and depletion of CD8^+^ T cells ablated the GNLY-mediated resistance to *P*. *yoelii*. Altogether, our results indicate that in addition to previously described mechanisms, IFN-γ promotes host resistance to the Retic-tropic *P*. *yoelii* 17XNL strain by promoting MHC-I expression on iRetics that become targets for CD8^+^ cytotoxic T lymphocytes and GNLY.

## Author summary

CD8^+^ cytotoxic T lymphocytes (CTLs) are important for immune defense against intracellular pathogens, such as viruses, bacteria and parasites, and tumor surveillance. CTLs, which recognize peptide epitopes presented by MHC-I molecules expressed in nucleated cells, become activated and kill infected target cells by releasing the contents of cytotoxic granules into the immunological synapse. Since most *Plasmodium spp*. infect erythrocytes that are enucleated and do not express MHC-I, the role of CD8^+^ T cells in the blood-stage of malaria has been neglected. We recently showed that *P*. *vivax*-infected reticulocytes express MHC-I and are killed in a manner dependent on granulysin (GNLY), a cytotoxic granule effector protein. However, the protective role of CD8^+^ T cells is controversial and the role of GNLY *in vivo* remains to be demonstrated. Here, we show that CTLs and GNLY mediate mouse resistance to blood-stage infection with *P*. *yoelii*, a rodent malaria parasite that preferably infects reticulocytes.

## Introduction

*Plasmodium* infection remains a serious public health problem, causing over 200 million cases and ~435,000 deaths each year [1]. The lack of an effective vaccine and increasing drug resistance exacerbate the problem. The complex immune response of the host to the malaria parasite is not fully understood. Different *Plasmodium* species/strains vary substantially in the immune response they induce. In particular, the parasite preference for invading red blood cells (RBCs) at different stages of maturation may contribute to disease pathogenesis [2].

*Plasmodium vivax* exclusively infects reticulocytes (Retics), while *Plasmodium falciparum* primarily infects mature RBCs [2]. Retics are immature RBCs produced in the bone marrow by enucleation of the precursor erythroblast [3]. Retics continue to synthesize proteins, since they retain RNA, ribosomes and mitochondria. As they mature, Retics gradually lose the protein synthesis machinery as they develop into mature erythrocytes [4]. Although most circulating Retics express undetectable MHC, we recently reported that *P*. *vivax*-infected Retics (iRetics) express high levels of HLA class I (HLA-I) on their surface, comparable to B lymphocytes [5]. HLA-I expression enabled CD8^+^ T lymphocytes from *P*. *vivax*-infected patients to recognize, become activated and lyse iRetics, kill the parasite inside and prevent it from infecting new Retics. Recognition of iRetics also stimulated CD8^+^ T cells to secrete IFN-γ. iRetic lysis and parasite killing depended on CD8^+^ T cell release of cytotoxic granules and their contents, especially the death-inducing proteases (granzymes) and an antimicrobial peptide granulysin (GNLY), which preferentially permeabilizes cholesterol-poor membranes of bacteria, fungi and parasites rather than host cell membranes [6]. Unlike *P*. *vivax* iRetics, *P*. *falciparum*-infected RBCs do not express HLA-I, are not recognized by patient CD8^+^ T cells (manuscript in preparation) and are not thought to contribute to immune protection in erythrocytic *P*. *falciparum* infection [7–9].

Cytotoxic CD8^+^ T lymphocytes (CTLs) have multiple functions, in addition to killing by cytotoxic granule release, they can kill by engaging death receptors, such as Fas, on the surface of target cells and can release cytokines, especially IFN-γ and TNF-α, when they are activated by recognizing a target cell [10]. The *in vivo* role of CD8^+^ T lymphocyte recognition of iRetics and GNLY in protection from blood-stage *P*. *vivax* malaria is difficult to study in humans. Since *P*. *yoelii* 17XNL preferentially infects Retics [11], it is a good mouse model to study the role of CD8^+^ T cells in host defense against Retic-tropic *Plasmodium* species. However, some key immune receptors, such as some of the NK receptors, which are also expressed on activated CD8^+^ T cells differ substantially between mice and humans. An important caveat is that mice and other rodents, unlike most mammals, do not express GNLY, which plays a critical role in parasite control by CD8^+^ T lymphocytes *in vitro* [12]. The role of GNLY, however, can be studied in mice by comparing wild-type (WT) mice with mice expressing a *GNLY* transgene (*GNLY*-Tg), in which GNLY is expressed only in cytotoxic lymphocytes, as in humans [13].

Several studies have evaluated the contribution of CTLs to host resistance to blood-stage *P*. *yoelii* in rodent malaria models. Although some studies suggest a protective role for CTLs during blood-stage infection, there are also conflicting results [14–22]. It should be emphasized that studies by Imai et al. (2013; 2015) focused on the activation of CTLs by parasitized erythroblasts that are nucleated cells and naturally express MHC-I. None of these mouse models expressed *GNLY*.

Because the contribution of CTLs in the *in vivo* immune response during blood-stage Retic-tropic malaria remains controversial, here we investigated whether and how CD8^+^ T cells control *P*. *yoelii* infection. Our results indicate that CTL-mediated resistance to blood-stage malaria requires IFN-γ-dependent expression of MHC-I on iRetics and is partially mediated by GNLY.

## Results

### *P*. *yoelii* induces reticulocytosis and preferentially infects reticulocytes

**Fig 1:**
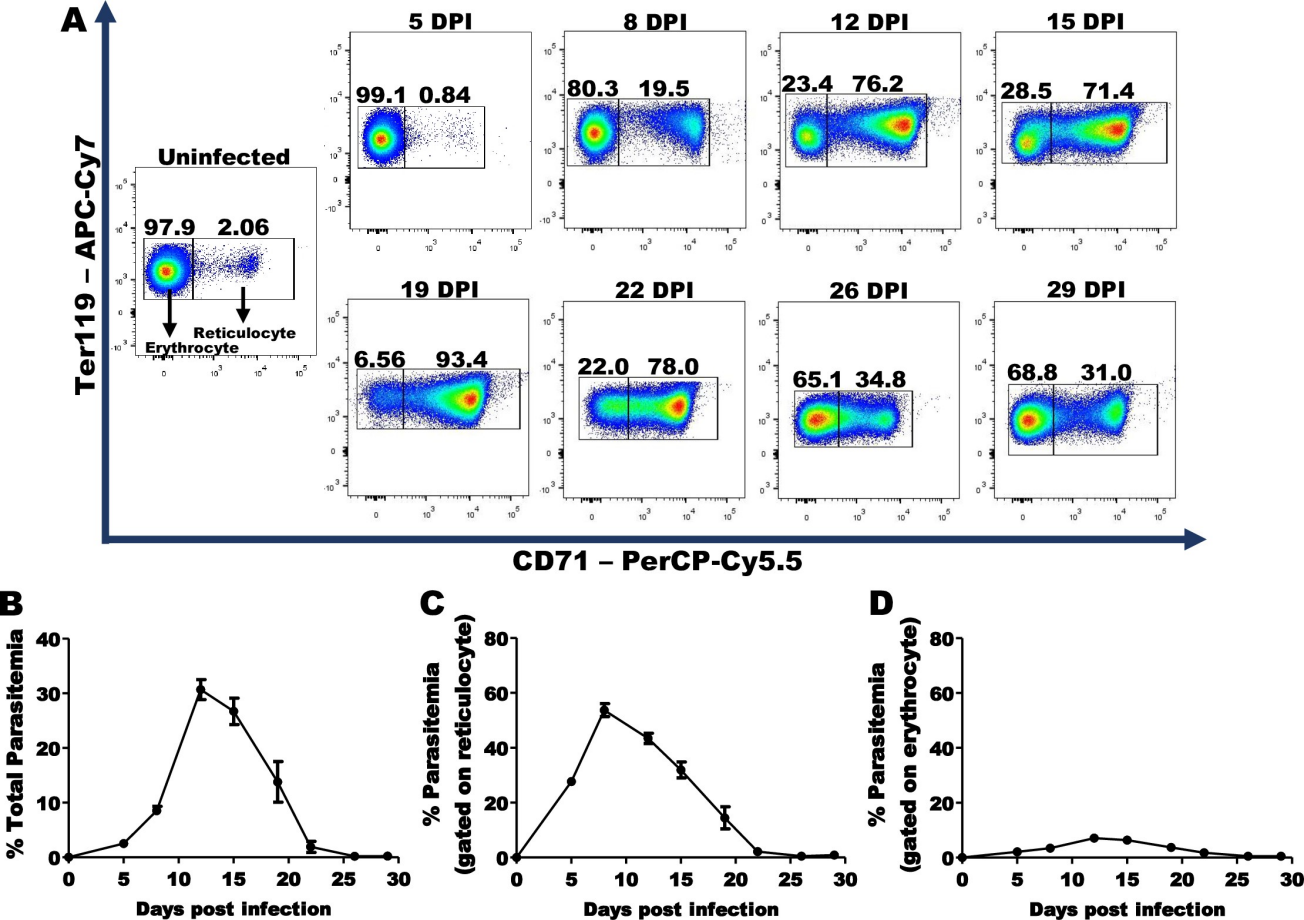
Reticulocytosis and parasitemia in *P*. *yoelii*-infected mice. **(A)** Representative dot-plots at different days post infection (DPI) show a dramatic increase of circulating Retics in the bloodstream of *P*. *yoelii*-infected mice. **(B)** Parasitemia curve shows the presence of *P*. *yoelii* within 30% of RBCs (12DPI). Parasitemia gated on Retics **(C)** and mature erythrocytes **(D)** shows that *P*. *yoelii* preferentially infects Retics. Data are pool of four independent experiments (n = 24).

WT mice were infected with *P*. *yoelii* 17XNL, a non-lethal GFP^+^ *Plasmodium* strain that is tropic for Retics. The frequency of circulating Retics and parasitemia were assessed for 30 days post-infection (DPI) by gating on cells stained for Ter119 and CD71, murine erythroid progenitor markers [23] (S1A Fig). Although uninfected mice had only a few percent Retics in their blood, reticulocyte counts increased beginning within a week and peaked 19 DPI when they constituted >90% of RBCs (Fig 1A). The peak of parasitemia occurred at 12 DPI when 30% of RBCs were parasitized (Fig 1B). About 50% of Retic were parasitized at 8 DPI (Fig 1C), but only a small proportion (< 7%) of mature erythrocytes were infected with *P*. *yoelii* at any time (Fig 1D). Parasitemia cleared around 26 DPI (Fig 1B). The total number of RBCs decreased during *P*. *yoelii* infection and returned to normal levels after the parasite clearance (S1B Fig). Therefore, there is a reduction of erythrocytes during the infection inversely to the increase in Retics (S1C and S1D Fig).

### Extramedullary hematopoiesis in *P*. *yoelii*-infected mice

**Fig 2:**
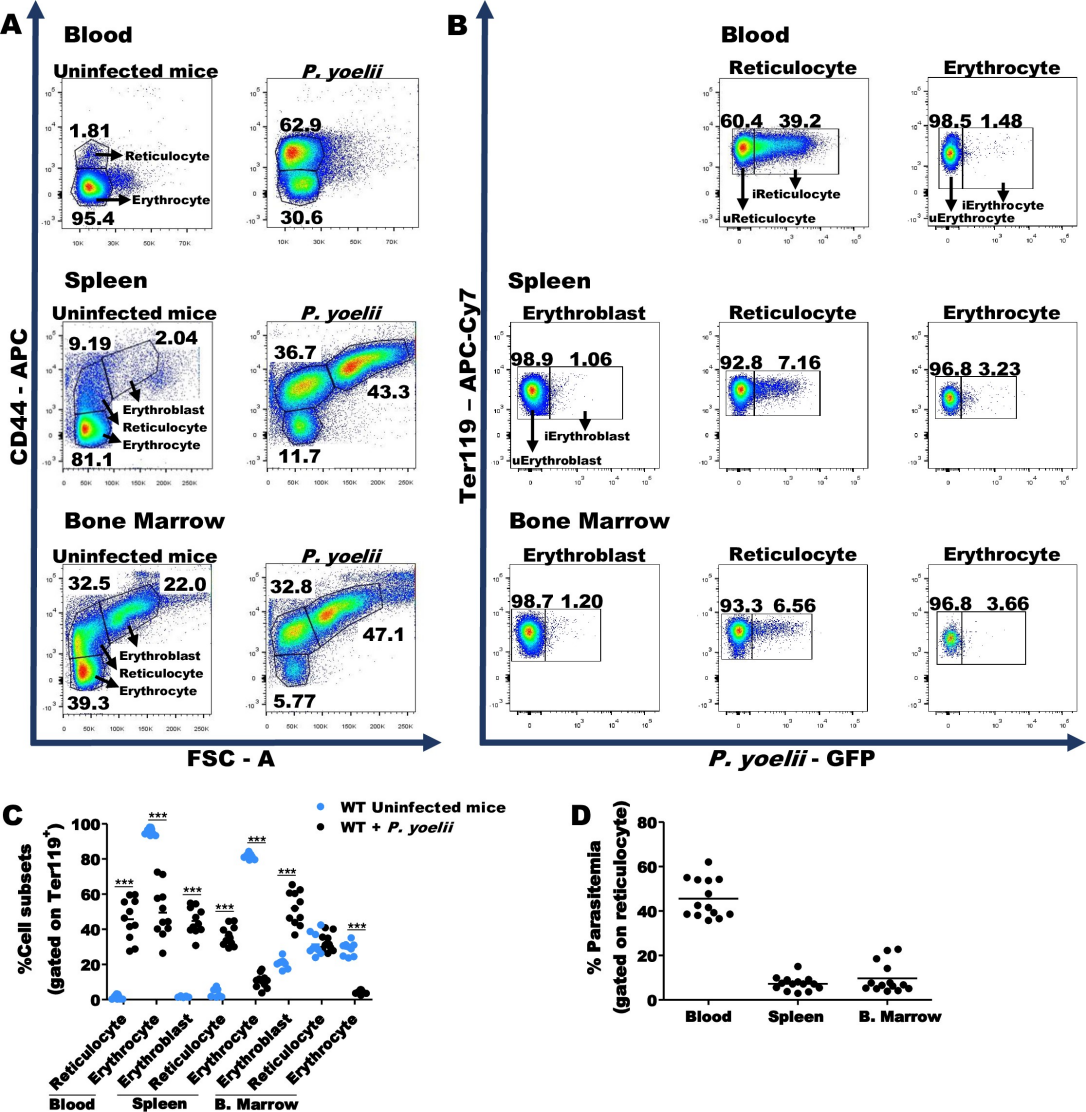
*P*. *yoelii* infection induces extramedullary erythropoiesis. **(A)** Representative dot-plots show the frequency of erythroblasts, Retics and mature erythrocytes in blood, spleen and bone marrow from uninfected mice and *P*. *yoelii*-infected mice 12 DPI. The RBCs were pre-gated on Ter119^+^. *P*. *yoelii*-infected mice exhibit erythropoiesis in spleen and bone marrow (middle and lower panels). **(B)** Evaluation of RBCs subsets during *P*. *yoelii* infection (black circles) shows higher frequency of Retics in blood and spleen, associated with increased erythroblasts in spleen and bone marrow in comparison to uninfected mice (blue circles). **(C)** *P*. *yoelii*-infected Retics can be found in blood, spleen or bone marrow. **(D)** However, the highest percentage of iRetics is found in the blood. The statistical analysis of cell subsets was performed using unpaired t-test or Mann-Whitney U test, according to data distribution. Data are pooled from three independent experiments (n = 9, uninfected mice; n = 11, *P*. *yoelii*-infected mice) ***p<0.001.

To evaluate the tissues where infection occurs, we compared infection on RBC lineage cells in blood, bone marrow (BM) and spleen 12 DPI (Fig 2A). RBCs at different maturation stages were identified by gating on CD44 and FSC [24,25] (S2A Fig). The percentage of erythroblasts in the BM increased by ~2-fold with infection (Fig 2A, lower panel and 2B). In addition, a high frequency of erythroblasts and Retics were found in the spleens of infected mice, indicating that this lymphoid organ had become an erythropoietic organ (Fig 2A, middle panel and 2B). On average, more than 40% of splenic RBCs were erythroblasts and 35% were Retics, while only a few percent of splenic RBCs were erythroblasts or Retics in uninfected mice. We also evaluated *P*. *yoelii* 17XNL infection in these RBC cell subsets in each compartment. iRetics was observed in the three compartments (blood, BM and spleen). At the peak of parasitemia, almost half of blood Retics were infected, while on average fewer than 10% of splenic or BM Retics were infected with *P*. *yoelii*. There were no infected BM or splenic GFP^+^ erythroblasts compared to background and <5% of mature RBCs were GFP^+^ in infected mice (Figs 2C and S2B and S2C). Thus, Retics are the dominant infected cells and iRetics can be found in BM, spleen and blood (Fig 2D). Our results are consistent with previous reports [21,26,27] showing that RBC precursors can be infected by *P*. *yoelii* in the BM of mice as in *P*. *vivax*-infected individuals.

### Expression of MHC class I on reticulocytes from *P*. *yoelii* infected mice

**Fig 3:**
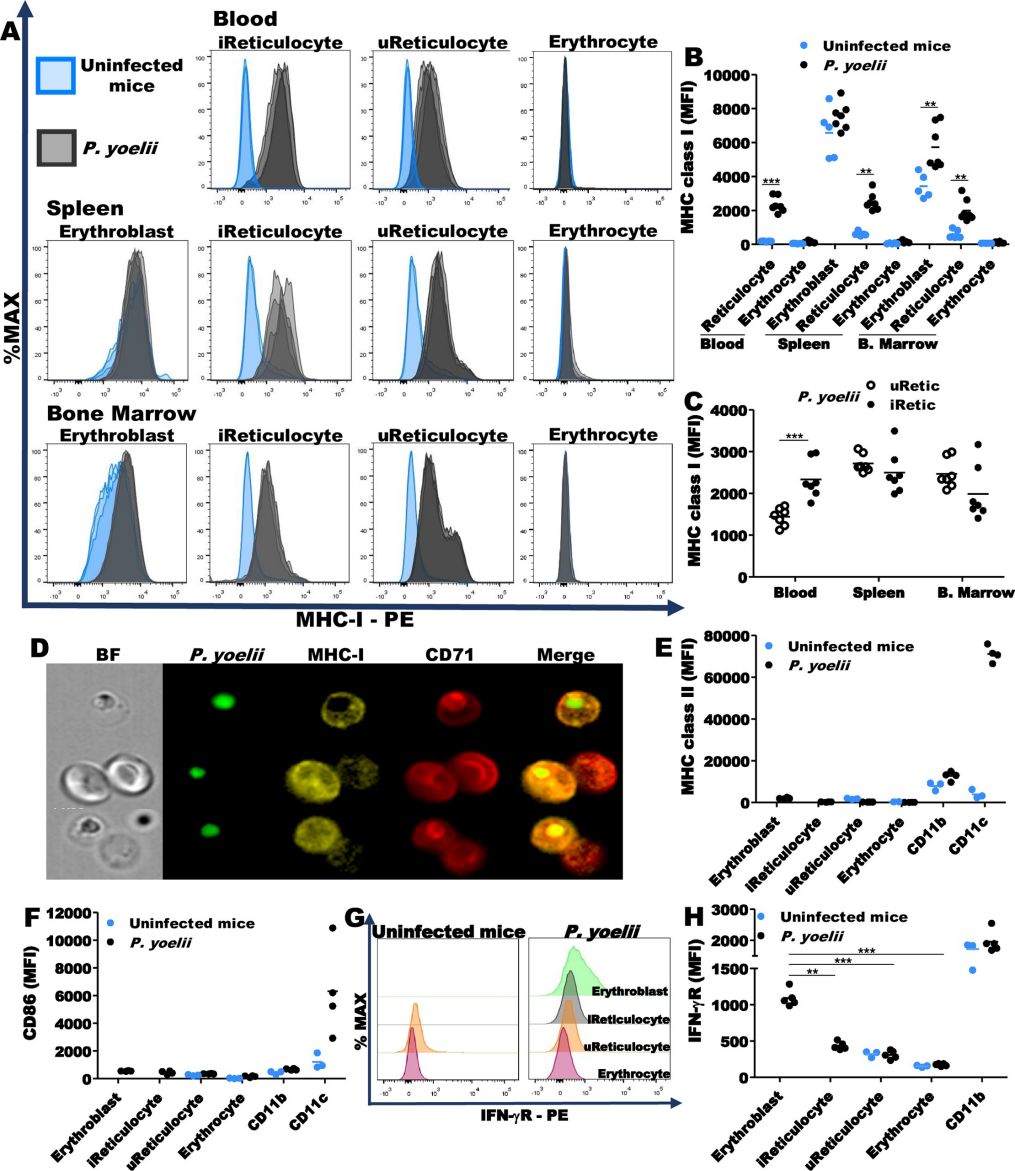
*P*. *yoelii*-infected Retics express MHC class I. **(A)** Representative histograms of MHC-I expression on RBCs at different stages of maturation in uninfected mice (blue) and *P*. *yoelii*-infected mice (black) at 12DPI. **(B)** *P*. *yoelii*-infected Retics (iRetics) from blood, spleen and bone marrow express MHC class I on their surface. Data are pooled from two independent experiments (n = 5, uninfected mice; n = 7, *P*. *yoelii*-infected mice). **(C)** iRetics (filled circles) from bloodstream express higher levels of MHC-I than uninfected Retics (uRetics; open circles) from *P*. *yoelii*-infected mice. **(D)** Imaging flow cytometry of Retics purified from the blood of *P*. *yoelii*-infected mice confirm the difference in MHC-I expression between iRetics and uRetics. **(E, F)** Circulating RBCs do not express MHC class II or CD86 in their surface (n = 3, uninfected mice; n = 4 *P*. *yoelii*-infected mice). **(G, H)** Erythroblasts express IFN-γR on their surface (n = 3, uninfected mice; n = 5 *P*. *yoelii*-infected mice). Statistical analysis of MHC-I and IFN-γR expression was carried out using unpaired t-test or Mann-Whitney U test, according to the data distribution. **p<0.01; ***p<0.001.

We next investigated whether *P*. *yoelii* 17XNL, like *P*. *vivax*, iRetics express cell surface MHC class I (MHC-I). RBCs at different stages of maturation were analyzed 12 DPI. As expected, erythroblasts expressed MHC-I independently of infection, (Fig 3A, left panels). However, BM erythroblasts from infected mice expressed significantly more MHC-I than erythroblasts from uninfected mice (Fig 3B). Although Retics from uninfected mice barely expressed MHC-I, Retics from infected mice expressed MHC-I, regardless of their location or whether they were infected (Figs 3A, middle panels and 3B). Moreover, iRetics in the blood, but not the BM or spleen, expressed significantly more MHC-I if they were infected [2,334±172.7 iRetic vs 1,440±80.4 uninfected Retic (uRetic), p = 0.0005] (Fig 3C). However, iRetics expressed about a log less MHC-I than splenic lymphocytes (S3A Fig). iRetics also expressed more MHC-I than uRetics from infected mice by imaging flow cytometry (15,256±525.8 iRetic vs 8,614±137.3 uRetic, p<0.0001; Fig 3D, middle and lower panels). The percentage of infected mature RBCs was very low (Fig 2C) and these cells did not express MHC-I in any compartment (Fig 3A, right panels and 3B). As expected, RBCs at different stages of maturation did not express MHC class II (Fig 3E) or CD86 (Fig 3F), a costimulatory molecule expressed by professional antigen presenting cells. Thus, uRetics and iRetics from *P*. *yoelii*-infected mice express MHC-I, but not class II, in all compartments. This result differs from *P*. *vivax*-infected humans in whom only iRetic, but not uRetic, express HLA-I at levels comparable to lymphocytes. Considering the involvement of IFN-γ in the MHC-I expression, we decided to evaluate the presence of IFN-γ receptor (IFN-γR) on the RBCs surface at different stages of maturation. Erythroblasts express IFN-γR on their surface and a residual expression can be seen in iRetics. On the other hand, erythrocytes do not express IFN-γR (Figs 3G, 3H and S3B). The evaluation at different time points showed that the MHC-I expression on Retic surface begins to be observed around 5DPI, being more pronounced in iRetic than in uRetic (S3C-D). Importantly, the emergence of MHC-I on Retic surface concurred with the increase in the number of IFN-γ-producing T cells, suggesting the participation of IFN-γ in this process (S3E).

### MHC class I expression by Retics is IFN-γ-dependent

**Fig 4:**
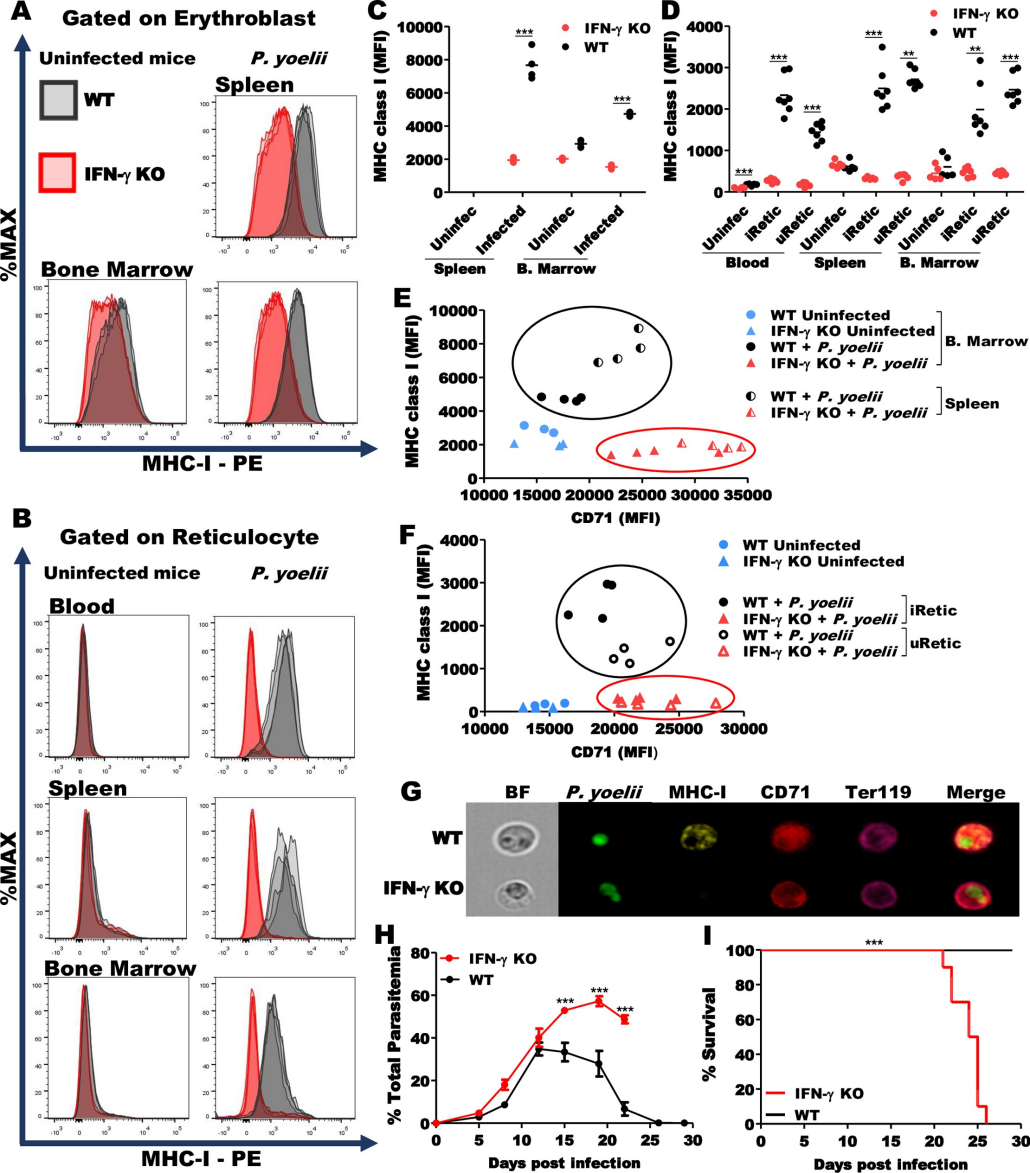
MHC class I expression on erythroblasts and Retics depends on IFN-γ. **(A, B)** Representative histograms of MHC-I expression on erythroblasts and Retics from *P*. *yoelii*-infected WT (grey) and IFN-γ KO (red) mice at 12 DPI. **(C)** Erythroblasts from spleen and bone marrow of *P*. *yoelii*-infected WT (black circle) express higher levels of MHC-I in comparison to IFN-γ KO (red circles) mice. Note that the frequency of erythroblasts in spleen of uninfected mice is below the limit of detection. **(D)** iRetics and uRetics from infected IFN-γ KO mice (red circles) express very low levels of MHC-I. **(E)** CD71 and MHC-I MFI correlation in erythroblast from uninfected mice (blue symbols) and *P*. *yoelii*-infected mice from bone marrow [filled black (WT) and red (IFN-γ KO) symbols] and spleen [half open black (WT) and red (IFN-γ KO) symbols]. **(F)** Retics from uninfected mice (blue symbols) and iRetics [filled black (WT) and red (IFN-γ KO) symbols] and uRetics [open black (WT) and red (IFN-γ KO) symbols] from *P*. *yoelii*-infected mice. Note that MHC-I, but not CD71 expression is impaired both in erythroblasts and Retics from infected IFN-γ KO mice. **(G)** Imaging flow cytometry of iRetics purified from blood confirm the difference in MHC-I expression between WT and IFN-γ KO mice. **(H)** Comparison of percentage parasitemia in RBCs from IFN-γ KO mice (red line) and WT mice (black line). **(I)** All IFN-γ KO mice (red line) succumbed to *P*. *yoelii* infection between 21 and 26 DPI. The statistical analysis for MHC-I was performed using unpaired t-test or Mann-Whitney U test, according to data distribution. Data are pooled from two independent experiments (n = 3–5, uninfected mice; n = 4–7, *P*. *yoelii*-infected). The statistical analysis of parasitemia was carried out using two-way ANOVA followed by Bonferroni post-hoc test. The statistical analysis of survival was performed using the log-rank test. Data are pooled from two independent experiments (n = 12, for each group). **p<0.01, ***p<0.001.

Because IFN-γ potently induces expression of genes involved in antigen presentation, including MHC-I [28], we hypothesized that activated CTL secretion of IFN-γ might be responsible for inducing MHC-I on erythroblasts and Retics in infected mice. To investigate this hypothesis, we compared MHC-I expression on RBC precursors from *P*. *yoelii* 17XNL-infected WT and IFN-γ knockout (KO) mice. Erythroblasts, the precursor of Retics, are nucleated cells and express MHC-I regardless of infection. However, the infection induced an increase of MHC-I expression on splenic erythroblasts from WT mice, but not from IFN-γ KO mice (Fig 4A and 4C). Infection did not induce MHC-I in iRetic or uRetic in IFN-γ KO (Fig 4B and 4D). Erythroblasts from infected WT mice exhibited more than two-fold MHC-I on their surface in comparison to IFN-γ KO mice, being more striking in the spleen than in the bone marrow. Similar CD71 expression was observed in erythroblasts and Retics from WT, when compared to IFN-γ KO mice (Fig 4E). In both WT and IFN-γ KO mice, *P*. *yoelii* primarily infected young Retics, identified by high CD71 expression (Fig 4F). However, the Retics from IFN-γ KO mice, whether infected or not, did not show significant expression of MHC-I. The lack of MHC-I expression in iRetic from IFN-γ KO mice was also confirmed by imaging flow cytometry (Fig 4G). Importantly, the number of splenic and circulating Retics were decreased in the IFN-γ KO mice infected with *P*. *yoelii* (S4A–S4E Fig) by ~40%. Moreover, after the peak of parasitemia IFN-γ KO mice had significantly higher parasitemia than WT mice (Fig 4H) and by 20–26 DPI, all IFN-γ KO animals died, while all WT animals cleared the infection and survived (Fig 4I).

### Expression of IFN-stimulated genes (ISGs) by erythroblasts and Retics from mice infected with *P*. *yoelii*

**Fig 5:**
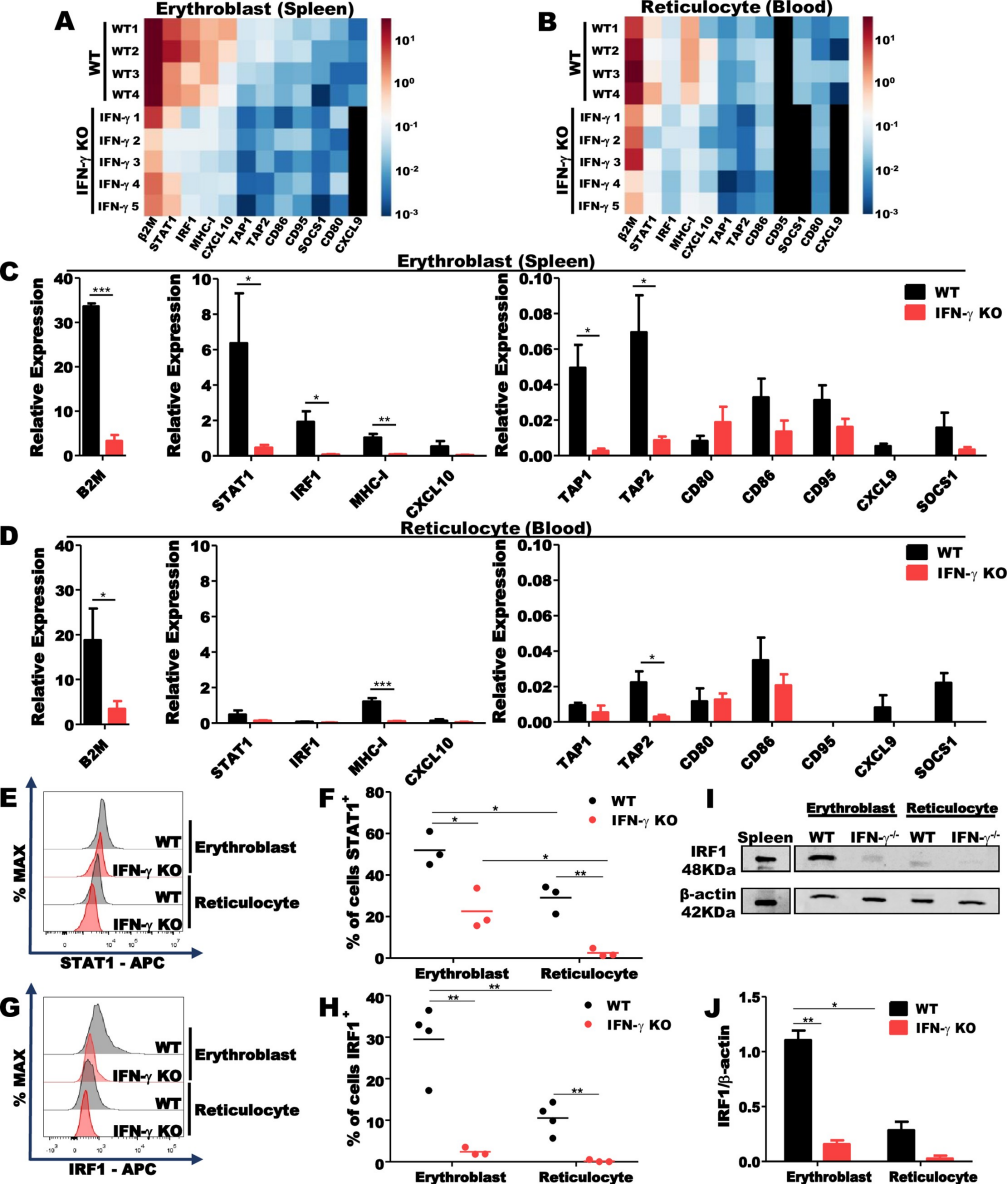
ISGs expression in erythroblasts and Retics from *P*. *yoelii* infected mice. **(A, B)** Heatmaps of ISGs expression on erythroblasts and Retics from WT and IFN-γ KO mice infected with *P*. *yoelii* at 12 DPI. **(C)** Splenic erythroblasts obtained from *P*. *yoelii*-infected WT (black bar) express high levels of *b2m*, *stat1*, *irf1*, *mhcI*, *cxcl10*, *tap1* and *tap2* mRNAs in comparison to IFN-γ KO (red bar) mice. **(D)** mRNA levels of *b2m*, *mhcI* and *tap2* remain high in Retics from peripheral blood of *P*. *yoelii*-infected WT (black bar), but not from IFN-γ KO mice (red bar). Data are pooled from two-three independent experiments (n = 4, WT mice; n = 5, IFN-γ KO). **(E)** Representative histogram of STAT1 levels in erythroblasts and Retics from *P*. *yoelii*-infected WT (grey) and IFN-γ KO (red) mice at 12 DPI. **(F)** Erythroblasts from spleen of *P*. *yoelii*-infected WT (black circle) express higher levels of STAT1 in comparison to infected IFN-γ KO (red circle) mice. A residual level of STAT1 can be observed in Retics from *P*. *yoelii*-infected WT (black circle) mice. **(G)** Representative histogram of IRF1 levels in erythroblasts and Retics from *P*. *yoelii*-infected WT (grey) and IFN-γ KO (red) mice at 12 DPI. **(H)** About 30% of erythroblasts from spleen of *P*. *yoelii*-infected WT (black circle) express IRF1 in comparison to 2.5% in IFN-γ KO (red circle) mice. Regarding the IRF1 levels in Retics, 10% of Retics from *P*. *yoelii*-infected WT (black circle) mice maintained the expression of IRF1 (n = 4, WT; n = 3 IFN-γ KO). **(I)** Western Blots of IRF1 in erythroblasts and Retics from WT and IFN-γ KO mice infected with *P*. *yoelii* at 12 DPI. **(J)** Erythroblasts from spleen of *P*. *yoelii*-infected WT (black bar) express higher levels of IRF1 in comparison to infected IFN-γ KO (red bar) mice. A residual level of IRF1 can be observed in Retics from *P*. *yoelii*-infected WT (black bar) mice. The statistical analysis was performed using unpaired t-test or Mann-Whitney U test, according to data distribution. *p<0.05; **p<0.01; ***p<0.001.

We next investigated the expression of ISGs in erythroblasts and Retics from WT and IFN-γ knockout (KO) mice infected with *P*. *yoelii* 17XNL. Increased expression *b2m*, *stat1*, *irf1*, *mhci*, *tap1* and *tap2* genes was observed in splenic erythroblasts from infected WT, but not from IFN-γ KO mice (Fig 5A and 5C). Similar results were obtained when we analyzed expression of *b2m*, *mhci* and *tap2* genes in circulating Retics (Fig 5B and 5D). Thus, IFN-γ is needed to induce expression of *b2m*, *mhci*, *tap1* and *tap2* genes that are involved in the presentation of endogenous antigens. Of note, IRF-1 is an important transcription factor that induces expression of MHC-I and other ISGs. Surprisingly, we found that the levels of β2-microglobulin and MHC-I mRNAs were similar in erythroblasts and reticulocytes. These results suggest a higher stability of mRNAs encoding proteins of the MHC-I complex, which are, therefore, likely to be synthesized in freshly enucleated reticulocytes.

The evaluation of STAT1 and IRF1 protein levels in erythroblast and Retics confirms the data obtained with the mRNA. Increased levels of STAT1 and IRF1 was observed in erythroblasts from infected WT, but not from infected IFN-γ KO mice (Figs 5E–5H and S4F and S4G). Residual levels of STAT1 and IRF1 were observed in Retics from infected WT mice, but not from infected IFN-γ KO mice (Figs 5E–5H and S4F and S4G). We also analyzed the levels of the IRF1 transcription factor by western blot. As expected IRF1 was increased in erythroblasts from infected WT mice (Fig 5I and 5J), reinforcing the data obtained by qPCR and flow cytometry.

### CD8^+^ T cells recognize *P*. *yoelii*-infected reticulocytes

**Fig 6:**
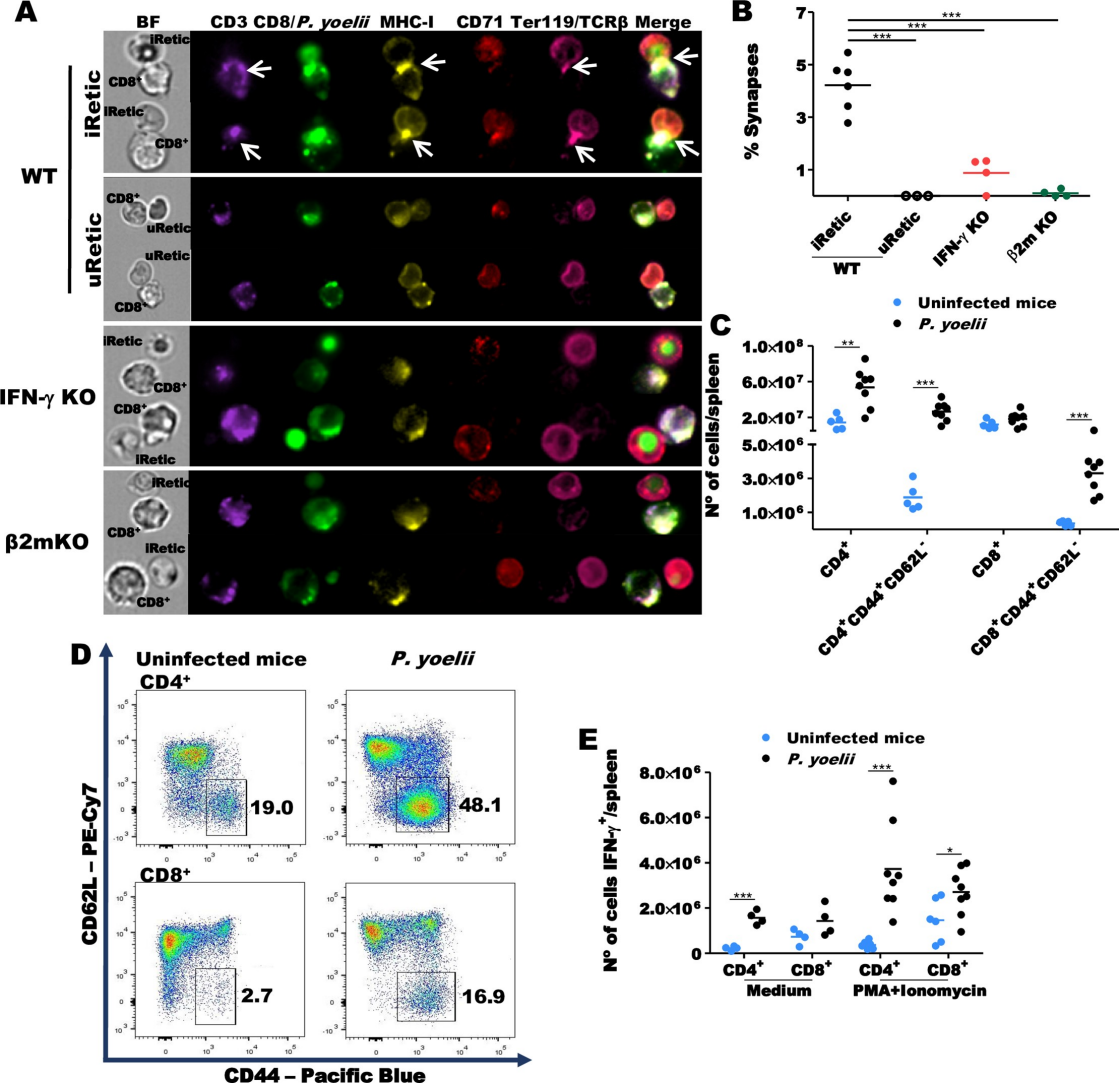
CD8^+^ T cells are activated and recognize iRetics during *P*. *yoelii* infection. **(A)** Imaging flow cytometry shows immunological synapses (arrows) between CD8^+^ T cells isolated from WT infected mice incubated with iRetics from WT infected mice (upper panels), but not with uRetics from WT and iRetics from IFN-γ KO mice (middle panels) or β2m KO mice (lower panels). **(B)** Percentage of CD8^+^ T cells from WT infected mice that formed immunological synapses with iRetics and uRetics purified from different strains of mice. **(C)** Representative dot-plots of activated CD4^+^ and CD8^+^ T cells in spleens of uninfected and infected WT mice 12 DPI. **(D)** *P*. *yoelii*-infected mice have more CD4^+^ T cells and activated CD4^+^ and CD8^+^ T cells in the spleen 12 DPI than uninfected mice. **(E)** More CD4^+^ T cells from *P*. *yoelii*-infected mice than from uninfected WT mice produce IFN-γ both with and without stimulation but the number of IFN-γ-producing CD8^+^ T cells is increased with infection only when stimulated. The statistical analysis of immunological synapse formation was carried out using one-way ANOVA followed by Bonferroni post-hoc correction. Data are pooled from two or three independent experiments (n = 6, WT; n = 4, IFN-γ KO and β2m KO). The statistical analysis of T cells was performed using unpaired t-tests and data are pooled from two independent experiments (n = 4–6, uninfected mice; n = 8, *P*. *yoelii*-infected mice). *p<0.05; **p<0.01; ***p<0.001.

To determine whether CD8^+^ T cells recognize iRetic, purified splenic CD8^+^ T cells from infected WT mice were incubated with iRetic and analyzed for immune synapse formation by imaging flow cytometry. CD8^+^ T cells formed conjugates with purified iRetics from WT mice in which MHC-I, TCRβ and CD3 all capped at an immune synapse (Fig 6A, upper panels and 6B). Τhe immunological synapse between CD8^+^ T cells and iRetics was often formed next to the parasitophorous vacuole within Retics. 70% of 146 synapses observed in WT samples had this feature. Despite the presence of MHC-I on uRetic surface, these cells did not form immune synapses with CD8^+^ T cells (Fig 6A, upper panels and 6B). In parallel experiments CD8^+^ T cells from infected WT mice were incubated with iRetics from IFN-γ KO or β2-microglobulin (β2-m) KO mice, which lack MHC-I expression in all cells. Very few capped synapses formed with iRetics from IFN-γ KO mice (Fig 6A middle and lower panels).

### CD8^+^ T cells are highly activated in *P*. *yoelii*-infected wild-type mice

We also evaluated the activation status of CD4^+^ and CD8^+^ T cells in the spleen of infected WT mice (Fig 6C and 6D). A significant increase in the number of splenic CD4^+^ T cells, but not CD8^+^ T cells was observed during *P*. *yoelii* infection (Fig 6D). The number of both CD4^+^ T and CD8^+^ T splenocytes that express CD44^+^CD62L^-^, a marker of effector and effector memory cells, increased dramatically (CD4^+^: 2.65x10^7^±3.66x10^6^ *P*. *yoelii* vs 1.89x10^6^±3.55x10^5^ uninfected mice, p = 0.0003; CD8^+^: 3.31x10^6^±4.30x10^5^ *P*. *yoelii* vs 3.56x10^5^±6.27x10^4^ uninfected mice, p = 0.0002) indicating that *P*. *yoelii* infection activated both T cell subsets (Fig 6D). There was no significant difference in the frequency and number of activated CD4^+^ T and CD8^+^ T lymphocytes, when comparing WT and IFN-γ KO mice infected with *P*. *yoelii* (S4 Fig).

The number of both CD4^+^ and CD8^+^ T cells from infected WT mice that produced IFN-γ after *ex vivo* stimulation with PMA and ionomycin also increased significantly (CD4^+^: 3.73x10^6^±7.20x10^5^ *P*. *yoelii* vs 3.73x10^5^±7.14x10^4^ uninfected mice, p = 0.0007; CD8^+^: 2.71x10^6^±3.69x10^5^ *P*. *yoelii* vs 1.46x10^6^±3.83x10^5^ uninfected mice, p = 0.04). In fact, the number of IFN-γ-producing CD4^+^ T cells was augmented in *P*. *yoelii* infection even without stimulation (Fig 6E).

### CD8^+^ T cells and GNLY contribute to *P*. *yoelii* control *in vivo*

**Fig 7:**
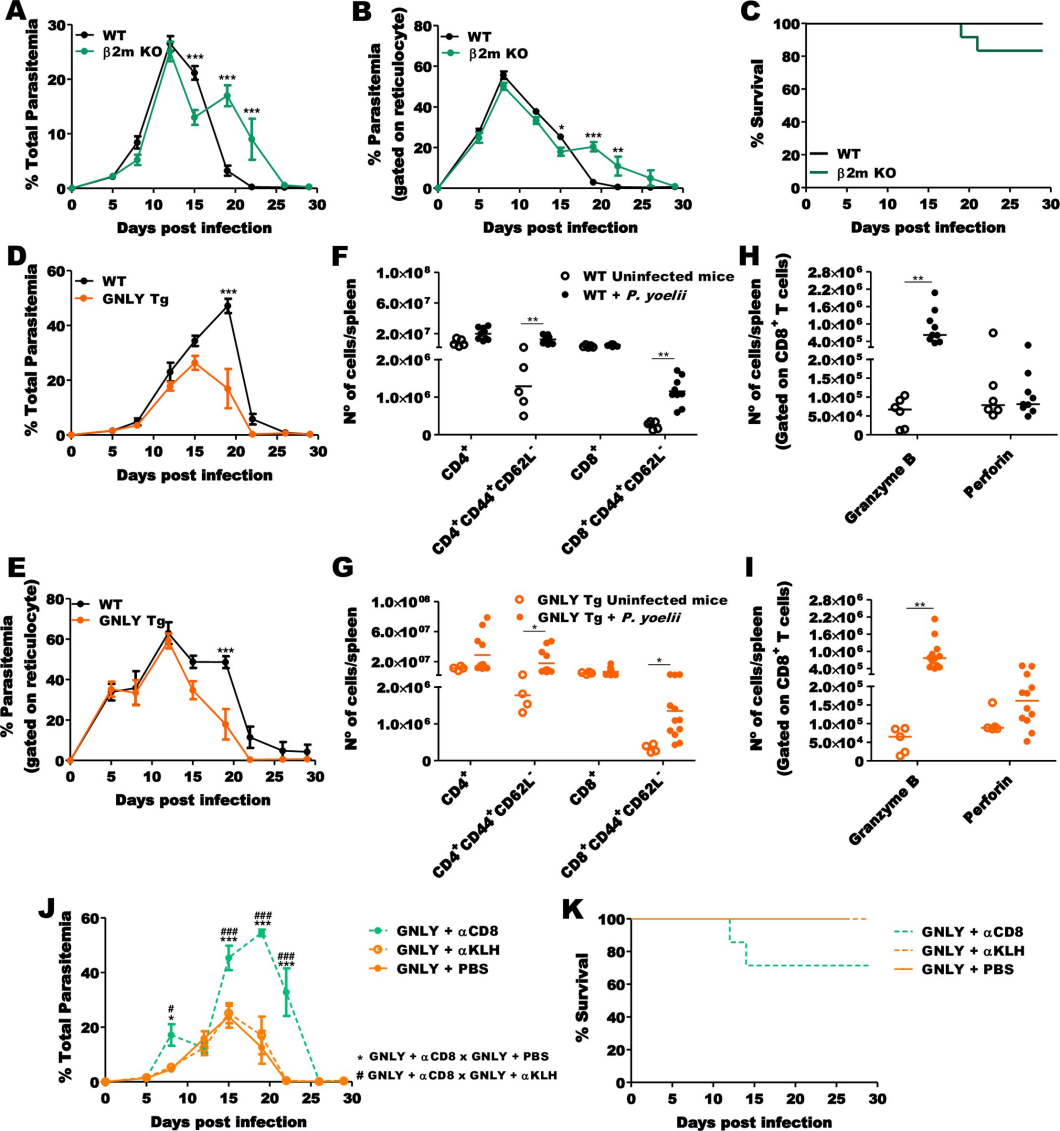
CD8^+^ T cells and GNLY contribute to *P*. *yoelii* infection control. Comparison of percentage parasitemia in RBCs **(A)** and Retics **(B)** in β2-m KO (green line) and WT mice (black line) shows the presence of a second peak of parasitemia around 19 DPI in β2-m KO mice. **(C)** 20% of β2-m KO mice (green line) die from *P*. *yoelii* but all WT mice (black) survive. Comparison of percentage parasitemia in RBCs **(D)** and Retics **(E)** in *GNLY*-Tg mice (orange line) and WT mice (black line) shows earlier control of parasitemia in *GNLY*-Tg mice compared to WT mice. *P*. *yoelii*-infected WT (**F and H)** and *GNLY*-Tg **(G and I)** mice have more activated T cells and higher expression of granzyme B in CD8^+^ T cells than uninfected mice. *GNLY*-Tg mice treated with αCD8 have higher parasitemia 8 DPI and delayed parasite clearance **(J)** and reduced survival **(K)** compared to control *GNLY*-Tg mice that received αKLH or PBS. The statistical analysis of parasitemia was performed using two-way ANOVA followed by Bonferroni post-hoc test. The statistical analysis of survival was performed using a log-rank test. Data are pooled from two independent experiments (n = 7–12, for each group). The statistical analysis of T cells was performed using the Kruskal-Wallis test and data are pooled from two-three independent experiments (n = 4–6, uninfected mice; n = 9–12, *P*. *yoelii*-infected mice). *p<0.05; **p<0.01; ***p<0.001.

Finally, we evaluated the importance of CD8^+^ T cells and GNLY in host resistance to *P*. *yoelii* infection by comparing survival and parasitemia in WT, β2-m KO and *GNLY*-Tg mice. Because β2-m KO lack MHC-I expression in all cells (S4H Fig), they have no CD8^+^ T cells. The peak of parasitemia was similar in WT and β2-m KO mice. However, β2-m KO mice showed significantly higher parasitemia after the peak and delayed clearance, suggesting a role for malaria-specific CTLs, which take about a week after infection to develop, in controlling the infection (Fig 7A and 7B). In addition, unlike WT mice that all survived, 2 of 12 β2-m KO mice died around 20 DPI (Fig 7C).

When we compared *P*. *yoelii* infection in *GNLY*-Tg and WT BALB/c mice, the peak of parasitemia was ~2-fold lower and parasite clearance was also faster (Fig 7D and 7E). Although the number of splenic effector, effector memory and CTLs expressing GzmB or PFN were similar in *P*. *yoelii*-infected WT and *GNLY*-Tg mice (Figs 7F–7I and S5A and S5E). To confirm the importance of GNLY during *P*. *yoelii* infection, we depleted CD8^+^ T cells using a CD8 monoclonal antibody (S5F Fig). CD8-depleted *GNLY*-Tg mice exhibited higher parasitemia 8 DPI in comparison to control groups (treated with αKLH or untreated) (Fig 7J). Almost 30% of *GNLY*-Tg mice treated with αCD8 died around 14 DPI and parasitemia of surviving mice reached 60% 19 DPI when all WT mice had cleared the infection, confirming the importance of CD8^+^ CTLs in control of *P*. *yoelii* 17XNL infection (Fig 7K).

## Discussion

Here we investigated the role of IFN-γ, MHC-I expression on Retics, CD8^+^ T cells and GNLY in host resistance to the Retic-tropic *P*. *yoelii* 17XNL strain. MHC-I expression on iRetics depended on endogenous IFNγ. Unlike WT mice, IFN-γ KO mice had trouble clearing parasitemia and all died from a challenge that was not lethal to WT mice. CD8^+^ T cells from WT mice infected with *P*. *yoelii* blood-stage were highly activated and formed immune synapses with iRetics in an IFN-γ- and MHC-I-dependent manner. Finally, we found that the transgenic mice that express human GNLY, an anti-microbial peptide produced by CTLs, are more resistant to infection with *P*. *yoelii* blood-stage.

Earlier studies have shown that MHC-I (H-2K and H-2D) is expressed on the surface of *P*. *yoelii*-iRetics [15,29,30]. However, the results regarding the role of CD8^+^ T cells (Ly-2^+^ T cells) on host resistance to infection with the *P*. *yoelii* 17XNL blood-stage are conflicting [14–22]. Here, we confirm that Retics from *P*. *yoelii*-infected mice are MHC-I^+^. In particular, iRetics express high levels of MHC-I, corroborating that they are potential targets for CD8^+^ T cells. We also report that infection in WT mice greatly increases MHC-I expression on erythroblasts, the immediate precursor of the Retics that are infected by *P*. *yoelii*. Our data support the hypothesis that IFN-γ promotes expression of MHC-I on erythroblasts. Consistent with this hypothesis, the level of MHC-I expression on erythroblasts, both in the bone marrow and spleen, is not elevated in infected IFN-γ KO, contrasting with CD71 levels that remain high on erythroblasts or young Retics from *P*. *yoelii*-infected IFN-γ KO mice.

To further address this issue, we analyzed the expression of some ISGs. As expected, expression of ISGs was not enhanced in erythroblasts from infected IFNγ KO mice. Importantly, the expression of STAT1 and IRF-1 mRNA was highly augmented in erythroblasts, but not on Retics from infected WT mice. These findings were corroborated by assays that measured the levels of these proteins in erythroblasts and Retics. Of note, IRF-1 is an important transcription factor that promotes expression of MHC-I and other ISGs. Surprisingly, we found that levels of β2-m and MHC-I mRNAs remained high in Retics. These results suggest that while transcription of β2-m and MHC-I is induced in erythroblasts, translation and assembly of MHC-I complex may still occur in freshly enucleated Retics. In addition, reticulocytes in the infected mice are younger (*i*.*e*., less mature and CD71^high^) and on average spent less time since their transition from erythroblasts (enucleation) and therefore the time for MHC protein decay has been truncated, which should increase the MHC-I levels. Thus, our model allows us to conclude that IFNγ-induced expression of MHC I on young reticulocytes is indirect, from a transcriptional effect on erythroblasts, rather than directly promoting expression of MHC-I gene on recently enucleated red blood cells, which would be hard to explain mechanistically.

IFN-γ is a pleiotropic cytokine that has been implicated in enhancing erythropoiesis [31], MHC-I expression and presentation of endogenous antigens by infected host cells, increasing their susceptibility to killing by CTLs [28]. A recent study showed that *P*. *yoelii* growth is limited in IFN-γ KO mice, due to compromised erythropoiesis and a reduced number of circulating Retics [32]. We re-evaluated the importance of endogenous IFN-γ during infection with *P*. *yoelii* 17XNL strain. We found that infection with *P*. *yoelii* 17XNL induces splenic erythropoiesis and intense reticulocytosis, reaching the frequency of 90% of Retics in circulating RBCs. We confirm the results from Okada et al. (2015) showing that reticulocytosis is decreased in *P*. *yoelii*-infected IFN-γ KO mice (S5C Fig). However, in sharp contrast with Okada et al. (2015) findings, we report that IFN-γ KO mice infected with *P*. *yoelii* had increased parasitemia and succumbed to infection with a non-lethal strain of *P*. *yoelii* [33,34]. Importantly, Retics and iRetics from IFN-γ KO mice infected with *P*. *yoelii* did not express MHC-I. As a consequence, CD8^+^ T cells from infected WT mice did not form immunological synapses with iRetics from IFN-γ KO mice and β2-m KO mice that lack MHC-I expression. These findings support a potential role of CTLs in host resistance to infection. However, because IFNγ-KO mice are much more susceptible than β2-m KO, we speculate that IFNγ is acting in multiple ways to mediate resistance to infection with 17XNL strain. As B cell deficient mice are also highly susceptible to *P*. *yoelii* [35], IFN-γ-induced immunoglobulin switching may contribute to immune-mediated resistance to primary infection to *P*. *yoelii*. In addition, macrophages activated by IFN-γ may also contribute to phagocytosis and removing iRetics [36].

We next evaluated the main cellular sources of IFN-γ during infection with *P*. *yoelii* 17XNL. Earlier studies have shown changes in the frequency and number of resting and activated CD4^+^ T and CD8^+^ T cells during infection with non-lethal *P*. *yoelii* strain [37,38]. At the peak of parasitemia activated CD8^+^CD44^hi^CD62L^lo^ T cells seem to predominate [16]. We also evaluated the activation status of CD4^+^ and CD8^+^ T cells in spleens of *P*. *yoelii*-infected mice. On one hand, we observed an increase of the total number of CD4^+^ T cells, but not of CD8^+^ T cells. On the other hand, the frequency of both CD4^+^CD44^+^CD62L^-^ and CD8^+^CD44^+^CD62L^-^ T cells were increased at the peak of parasitemia. Both CD4^+^ T cells and CD8^+^ T cells were important producers of IFN-γ, with a small contribution of αβTCR^-^ cells.

The activation of CD4^+^ T cells in *P*. *yoelii*-infected mice was unanticipated because iRetics express MHC I, but not other co-stimulatory molecules or MHC II that are expressed by professional APCs and involved in antigen presentation and activation of naïve CD4^+^ T as well as CD8^+^ T cells. APCs in the secondary lymphoid tissues that phagocytose iRetics could activate CD4^+^ T cells in an antigen-specific manner, although if this were primary mechanism responsible for CD4^+^ T cell activation one would expect that it would be increased by IFN-γ. However, the level of CD4^+^ and CD8^+^ T cell activation was similar in *P*. *yoelii*-infected WT and IFN-γ KO, suggesting that IFN-γ did not contribute to T cell activation. An alternate explanation could be antigen-independent activation of bystander cells by pro-inflammatory cytokines or other interferons.

The role of CD8^+^ T cells in different rodent malaria models has been investigated. In the *P*. *berghei* ANKA rat model, both parasite clearance and anemia depend on CD8^+^ T cells [7]. Conflicting results have been obtained with the *P*. *chabaudi* strain [39,40] that primarily infects erythrocytes. Similarly, there is no consensus, regarding the role of CD8^+^ T cells, against *P*. *yoelii* 17XNL. In some studies, transfer and depletion experiments suggest a role for CD8^+^ T cells in host resistance to *P*. *yoelii* 17XNL [15,16,19–22]. However, experiments performed in other laboratories do not confirm these findings [14,17,18]. We addressed this question using the β2-m KO mice (CD8^+^ T cell-deficient mice) infected with *P*. *yoelii* 17XNL. In contrast with an earlier study [19], we observed a second peak of parasitemia and reduced survival in the β2-m KO, suggesting a role for CTLs in mouse resistance to *P*. *yoelii*. Similar findings were obtained in CD8-depleted mice infected with *P*. *chabaudi* AS that showed an intense reticulocytosis and two recurrent bouts of parasitemia, on day 16 (17,9%) and on day 30, (8,5%) leading to the conclusion that CD8^+^ T cells are important for parasite clearance [39,40]. Consistently, in a recent study, CD8^+^ T cells were shown to have a role in preventing relapse in mice chronically infected with *P*. *chabaudi* [41]. The rodents in all of these studies did not express GNLY, which limits their usefulness for understanding protective immunity in human malaria.

Finally, we tested the importance of human GNLY in mediating mouse resistance to *P*. *yoelii*-infection *in vivo*. The mouse genome does not encode *GNLY* [13]. Hence, we used a *GNLY*-transgenic mouse lineage. Importantly, our results show that *GNLY*-transgenic mice more efficiently control parasitemia and that depletion of CD8^+^ T cells ablates the protective effect of GNLY. Thus, our results suggest that GNLY expressed by CD8^+^ T cells contributes to the resolution of blood stage *P*. *yoelii* infection. These finding are in agreement with results indicating a protective role of GNLY to the experimental infection with either *T*. *cruzi* or *T*. *gondii* [12].

In conclusion, our results indicate that IFN-γ is essential for MHC-I expression on iRetics and that GNLY expressed by activated CD8^+^ T cells improves clearance of *P*. *yoelii* parasitemia. The present data support findings from our group showing that CTLs recognize and kill *P*. *vivax*-iRetics in a GNLY-dependent manner. These findings open up new perspectives for the investigation of CTL-based immune responses to the blood-stage malaria and the development of a vaccine that directly targets iRetics.

## Material and methods

### Animal experiments

WT C57BL/6 and BALB/c mice were purchased from Biotério Central UFMG (Belo Horizonte, MG). IFN-γ KO and β-2 microglobulin (β2-m) KO mice, originally from Jax Laboratories, were bred at Fiocruz-Minas animal facilities. *GNLY*-Tg mice on a BALB/c genetic background were obtained from JL lab (Boston Children’s Hospital, Boston, Massachusetts, USA) [12] and bred at Fiocruz-Minas animal facilities. Female and male mice, 8–10 weeks old, were used. Briefly, mice were infected with *P*. *yoelii* 17XNL PYGFP, a GFP-expressing strain purchased from MR4/ATCC (Manassas, VA). Cryopreserved parasites were thawed and passaged in mice twice before being used in experimental infections. A non-lethal inoculum of 10^5^ infected-RBCs was administered intraperitoneally (i.p.).

### CD8^+^ T cell depletion

*GNLY*-Tg mice were treated with 0.5 mg/mouse of rat anti-mouse CD8a mAb (clone 2.43, BioXCell) to deplete CD8^+^ T cells. Control *GNLY*-Tg mice were treated with 0.2 mg/mouse of rat anti-KLH IgG (clone LTF-2, BioXCell). Antibody treatment was administered i.p. on days -3, -2, -1 before infection and then every 7 d after infection. Depletion was confirmed by flow cytometry analysis of whole blood before infection.

### Parasitemia

A tail vein blood sample, diluted in PBS and Heparin (Cristália), was added to microtiter plates, washed with FACS Buffer (PBS + 2% FCS) and incubated with FcBlock (BD Bioscience) for 20 min at room temperature (RT). Cells were washed with FACS Buffer and stained with anti-Ter119 (APC-Cy7, BD Bioscience) and anti-CD71 (PerCP-Cy5.5, BD Bioscience). After 30 min incubation, cells were washed and the pellet resuspended in FACS Buffer. Flow cytometry was performed using BD LSRFortessa (BD Bioscience) and 100,000 RBCs were acquired and analyzed using FlowJo software (V10—Tri-Star).

### Flow cytometry

RBCs from peripheral blood, spleen and bone marrow were incubated with FcBlock (BD Bioscience) for 20 min at RT and then stained with anti-Ter119 (APC-Cy7, BD Bioscience), anti-CD71 (PerCP-Cy5.5, BD Bioscience or eFluor 450, Invitrogen), anti-CD44 (PE, APC or Pacific Blue, eBioscience), anti-CD86 (PE-Cy5, eBioscience), anti-MHC class I—H2Db (PE, Invitrogen), anti-MHC class II (APC, eBioscience), anti-IFN-γR (Biotin, BD Bioscience) and streptavidin (PE, eBioscience) for 30 min at RT. Cells were washed and the pellet resuspended in FACS Buffer. For intracellular staining of IRF1 and STAT1, the RBCs were fixed and permeabilized with Foxp3/Transcription Factor Staining Buffer Set (Invitrogen) according to the manufacturer’s instructions and blocked with Permeabilization Buffer supplemented with 2% of FCS for 45 min. The cells were stained with anti-IRF1 (Cell Signaling) or STAT1 (Abcam) for 30 min at RT, washed twice in Permeabilization Buffer and stained during 30 min at RT with secondary antibodies (Dnk anti-rabbit IgG, AF647—Abcan or Goat anti-mouse IgG, Cy5—Invitrogen). Flow cytometry was performed using BD LSRFortessa (BD Bioscience) and 100,000 RBCs were acquired. For T cell analysis, spleens were collected and WBCs were obtained after RBC lysis using ammonium chloride (150 mM). For *ex vivo* experiments, cells were resuspended in RPMI 1640 and plated at 2x10^6^ cells/well. Cells were then washed twice in PBS, stained with Live/Dead stain (Acqua, Invitrogen) for 20 min at 4°C in the dark, incubated with FcBlock (BD Bioscience) for 20 min at 4°C and then stained with anti-CD3 (PE-Cy5, BD Bioscience), anti-CD4 (Alexa Fluor 700, eBioscience), anti-CD8 (APC-Cy7, BioLegend), anti-CD44 (Pacific Blue, eBioscience) and anti-CD62L (PE-Cy7, BD Bioscience) for 30 min at 4°C. Cells were washed again and the pellet resuspended in FACS Buffer. To measure cytokine and granule proteins, cells were resuspended in RPMI 1640 supplemented with 10% FCS in the presence or absence of PMA (50 ng/mL) and ionomycin (500 ng/mL). Golgi Plug and Golgi Stop (BD Bioscience) were added according to the manufacturer’s instructions. Cells were cultured for 4 h at 37°C under 5% CO_2_, washed twice in PBS, stained with Live/Dead stain, incubated with FcBlock, and then stained with anti-CD3, anti-CD4 and anti-CD8, as described above. The cells were then permeabilized with Cytofix/Cytoperm (BD Bioscience) according to the manufacturer’s instructions and stained with anti-IFN-γ (APC, BD Bioscience) or anti-Granzyme B (eFluor450, eBioscience) and anti-Perforin (APC, Invitrogen). Flow cytometry was carried out using a BD LSRFortessa and ~100,000 live T cells were acquired, according to the Guidelines for the use of flow cytometry and cell sorting in immunological studies [42]. Data were analyzed using FlowJo software.

### Imaging flow cytometry

iRetics were purified from whole blood using a 50% Percoll gradient (GE Healthcare) and uRetics was obtained from the pellet. Cells were then stained with anti-Ter119 (APC-Cy7, BD Bioscience), anti-CD71 (PerCP-Cy5.5, BD Bioscience) and anti-MHC class I, H2Db (PE, Invitrogen) and incubated for 30 min at RT in the dark. Stained cells were washed and resuspended in saline buffer (0.9%) for image acquisition. For immunological synapse experiments, splenic CD8^+^ T cells were stained with anti-CD3 (eFluor450, eBioscience) and anti-CD8 (FITC, BD Bioscience) and purified by sorting on the BD FACSAria (BD Bioscience). Purified CD8^+^ T cells were stained with anti-TCRβ (APC-eFluor780, eBioscience) and incubated for 30 min at RT in the dark. Purified CD8^+^ T cells and iRetics or uRetics (purified and stained as described above) were co-cultured (1:5 ratio) for 2 h at RT with stirring. Image acquisition was performed using an ImageStream Imaging Flow Cytometer (Millipore) and the data were analyzed using Ideas software (Amnis).

### Quantitative reverse transcription (RT) PCR and Western Blot

Splenocytes were incubated with FcBlock (BD Bioscience) for 20 min at RT and then stained with anti-Ter119 (APC-Cy7, BD Bioscience), anti-CD71 (PerCP-Cy5.5, BD Bioscience) and anti-CD44 (APC, eBioscience) for 30 min at RT. Cells were washed, the pellet resuspended in saline buffer (0.9%) and erythroblasts were purified by sorting on the BD FACSAria (BD Bioscience). Retics were purified from whole blood using a 50% Percoll gradient (GE Healthcare).

For RNA isolation, the pellet was resuspended in Trizol Reagent (Invitrogen) and stored at -20°C until RNA extraction following manufacturer’s instructions. After total RNA isolation the samples were treated with DNase (Promega) and converted in cDNA using High-Capacity cDNA Reverse Transcription Kit (Applied Biosystems) according to manufacturer’s instructions. qPCRs reactions were carried out using Sybr Green PCR Master Mix (Applied Biosystems) in an ABI7500 Real Time PCR System (Applied Biosystems) under standard conditions. Primer sequences are presented in Table 1. qRT-PCR data were presented as 2^-ΔCt^.

**Table 1:** qPCR primer sequences.

Gene	Primer sequences	
β-actin	F 5’ CGATGCCCTGAGGCTCTTT 3’	R 5’ TGGATGCCACAGGATTCCAT 3’
β2M	F 5’ CCGAACATACTGAACTGCTACGTAA 3’	R 5’ CCCGTTCTTCAGCATTTGGA 3’
CD80	F 5’ GAGTCTGGAAACCCATCTGCA 3’	R 5’ GAAGCGAGGCTTTGGGAAAC 3’
CD86	F 5’ GGCCCTCCTCCTTGTGATG 3’	R 5’ CTGGGCCTGCTAGGCTGAT 3’
CD95	F 5’ ACTGCGATTCTCCTGGCTGT 3’	R 5’ TGGCTCAAGGGTTCCATGTT 3’
CXCL9	F 5’ AATGCACGATGCTCCTGCA 3’	R 5’ AGGTCTTTGAGGGATTTGTAGTGG 3’
CXCL10	F 5’ GCCGTCATTTTCTGCCTCA 3’	R 5’ CGTCCTTGCGAGAGGGATC 3’
GAPDH	F 5’ GGCAAATTCAACGGCACAGT 3’	R 5’ AGATGGTGATGGGCTTCCC 3’
HPRT	F 5’ CATAACCTGGTTCATCATCGC 3’	R 5’ GGAGCGGTAGCACCTCCT 3’
IRF1	F 5’ AGGCATCCTTGTTGATGTCC 3’	R 5’ AATTCCAACCAAATCCCAGG 3’
MHC-I	F 5’ AGTGGTGCTGCAGAGCATTACAA 3’	R 5’ GGTGACTTCACCTTTAGATCTGGG 3’
SOCS1	F 5’ ACAAGCTGCTACAACCAGGG 3’	R 5’ ACTTCTGGCTGGAGACCTCA 3’
STAT1	F 5’ TCACAGTGGTTCGAGCTTCAG 3’	R 5’ GCAAACGAGACATCATAGGCA 3’
TAP1	F 5’ GGACTTGCCTTGTTCCGAGAG 3’	R 5’ GCTGCCACATAACTGATAGCG 3’
TAP2	F 5’ CTGGCGGACATGGCTTTACTT 3’	R 5’ CTCCCACTTTTAGCAGTCCCC 3’

For western blot analysis, erythroblasts pellets were resuspended in RIPA Buffer (Sigma) containing protease and phosphatase inhibitor cocktail (cOmplete ULTRA and PhosSTOP, Roche). The Retics were treated with HemogloBind (Biotech Support Group) and the supernatant was collected and mixed with RIPA Buffer supplemented with inhibitor cocktail. The proteins in the supernatant were separated in SDS-PAGE and transferred to nitrocellulose membranes. The membranes were blocked with 5% of non-fat dry milk and incubated with primary antibodies anti-Jak (Abcam), anti-Stat1 (Abcam), anti-pStat1 (Abcam), anti-IRF1 (Cell Signaling) and anti-β-actin (Sigma). Following the membranes were incubated with secondary antibodies anti-rabbit (Sigma) or anti-mouse (Abcam) conjugated with HRP and revealed using the ECL system (Bio-Rad). The images were captured using an Amersham Imager 600 (GE). The data were analyzed using ImageJ software (NIH).

### Statistical analysis

Statistical analysis was conducted using Prism software 5.0 for Windows (GraphPad Inc, USA). Grubb’s test was applied to detect and remove possible outliers and the Kolmogorov-Smirnoff test was used to verify data distribution. Comparison between uninfected and infected groups was performed using unpaired *t*-test or Mann–Whitney U test, according to whether the data were normally distributed. Parasitemia was analyzed by two-way ANOVA followed by Bonferroni post-hoc test. For survival analysis, the log-rank test was used. Statistical differences were considered significant when *p* values ≤ 0.05.

### Ethics statement

All mouse experiments were performed according to the principles of conduct of the Brazilian Practice Guide for the Care and Use of Animals for Scientific and Didactic Purposes of CONSEA (http://www.sbcal.org.br/). The protocols were approved by the Fiocruz Council of Animal Experimentation (CEUA protocol LW17/18).

## Supporting information

S1 Fig Gating strategy and RBCs count. **(A)** Gating strategy for the analysis of parasitemia. RBCs count during *P*. *yoelii* infection, presented as total RBCs **(B),** Retics **(C)** and erythrocytes **(D).**
(TIF)

S2 Fig Flow cytometry gating strategy for RBC subsets and control background for GFP^+^ *P. yoelii* from uninfected mice. **(A)** Gating strategy for the analysis of RBC subsets and GFP^+^ *P*. *yoelii*-infected cells within these subsets. **(B)** Representative dot-plots show the frequency of erythroblasts, Retics and erythrocytes in blood, spleen and bone marrow from uninfected mice. **(C)** Evaluation of GFP^+^ control background in RBCs subsets from uninfected mice.
(TIF)

S3 Fig MHC class I and IFN-γR expression and IFN-γ production during *P. yoelii* infection. **(A)** The expression of MHC-I on Retics and lymphocytes (magenta) was compared between uninfected mice (blue) and *P*. *yoelii*-infected mice (grey). iRetics showed higher levels of MHC-I compared to Retics from uninfected mice. However, iRetics express about a log less MHC-I than splenic lymphocytes. Similar levels of MHC-I expression were observed in lymphocytes regardless of infection. **(B)** Representative histograms of IFN-γR in RBCs subsets and CD11b^+^ from uninfected and *P*. *yoelii*-infected mice at 12 DPI. Purple histogram represent background (sample stained with streptavidin only). **(C)** Representative histograms of MHC-I expression on iRetics and uRetics from blood of infected mice at 3, 5 and 10DPI. **(D)** iRetics (filled circles) express MHC-I on their surface earlier than uRetics (open circles) in *P*. *yoelii*-infected mice. **(E)** The number of IFN-γ-producing T cells increases during the course of infection and concur with emergence of MHC-I on Retics surface.
(TIF)

S4 Fig RBCs and T cells status in IFN-γ KO and WT mice during *P. yoelii* infection. *P*. *yoelii*-infected mice show an increase in the percentage of reticulocytes in the blood and spleen both in WT **(A)** and IFN-γ KO **(B)** mice. However, reticulocytosis is less pronounced in IFN-γ KO infected mice **(C)**. **(D)** The number of CD4^+^ and CD8^+^ T cells expressing activation markers (CD44^+^ and CD62L^-^) during *P*. *yoelii* infection is increased both in WT (black circles) and IFN-γ KO (red circles) mice. **(E)** *P*. *yoelii*-infected IFN-γ KO mice (filled red circles) do not increase the number of CD8^+^ T cells producing granzyme B unlike infected WT mice (filled black circles). However, the number of CD8^+^ T cells producing perforin is increased in IFN-γ KO mice (filled red circles) 12 DPI. Representative dot-plots of STAT1 **(F)** and IRF1 **(G)** levels in *P*. *yoelii*-infected WT (grey) and IFN-γ KO mice (red) at 12 DPI. The green histograms represent background (samples stained with secondary antibody only). **(H)** β2-m KO mice (green) do not express MHC-I. Representative histograms of Retics from blood, spleen and bone marrow. Retics and lymphocytes were isolated from uninfected mice or *P*. *yoelii*-infected mice at 12 DPI. The statistical analysis of RBCs subsets was performed using unpaired t-test or Mann-Whitney U test, according to the normality of data distribution. Data are pooled from two-three independent experiments (WT: n = 9, uninfected mice; n = 11, *P*. *yoelii*-infected mice; IFN-γ KO: n = 5, uninfected mice; n = 10, *P*. *yoelii*-infected mice). The statistical analysis of T cells was performed using unpaired t-test or Mann-Whitney U test, according to data distribution. Data are pooled from two independent experiments (n = 8 for each groups). **p<0.01; ***p<0.001.
(TIF)

S5 Fig Representative dot-plots of T cells from WT and *GNLY*-Tg mice. **(A)** Dot plots of CD4^+^ and CD8^+^ T cell subsets from WT (upper panels) and *GNLY*-Tg (lower panels). Activation status dot plots of CD4^+^ T **(B)** and CD8^+^ T **(C)** cells from WT (upper panels) and *GNLY*-Tg (lower panels). Granzyme B **(D)** and Perforin **(E)** dot plots of splenic CD8^+^ T cells in WT (upper panels) and *GNLY*-Tg (lower panels). Analyses were performed using splenocytes from uninfected or *P*. *yoelii*-infected mice 12 DPI. **(F)** Dot plot of CD4^+^ and CD8^+^ T cells subsets from *GNLY*-Tg mice depleted of CD8^+^ T cells (left panel) and *GNLY*-Tg mice treated with αKLH (right panel). This analysis was performed 3 days post antibody treatment.
(TIF)
