# Supplementary figures and images for: Contributions of IFN-γ and granulysin to the clearance of Plasmodium yoelii blood stage
Source: PLoS Pathog. 2020 Sep 10;16(9):e1008840. doi: 10.1371/journal.ppat.1008840 (PMC7482970; doi:10.1371/journal.ppat.1008840)

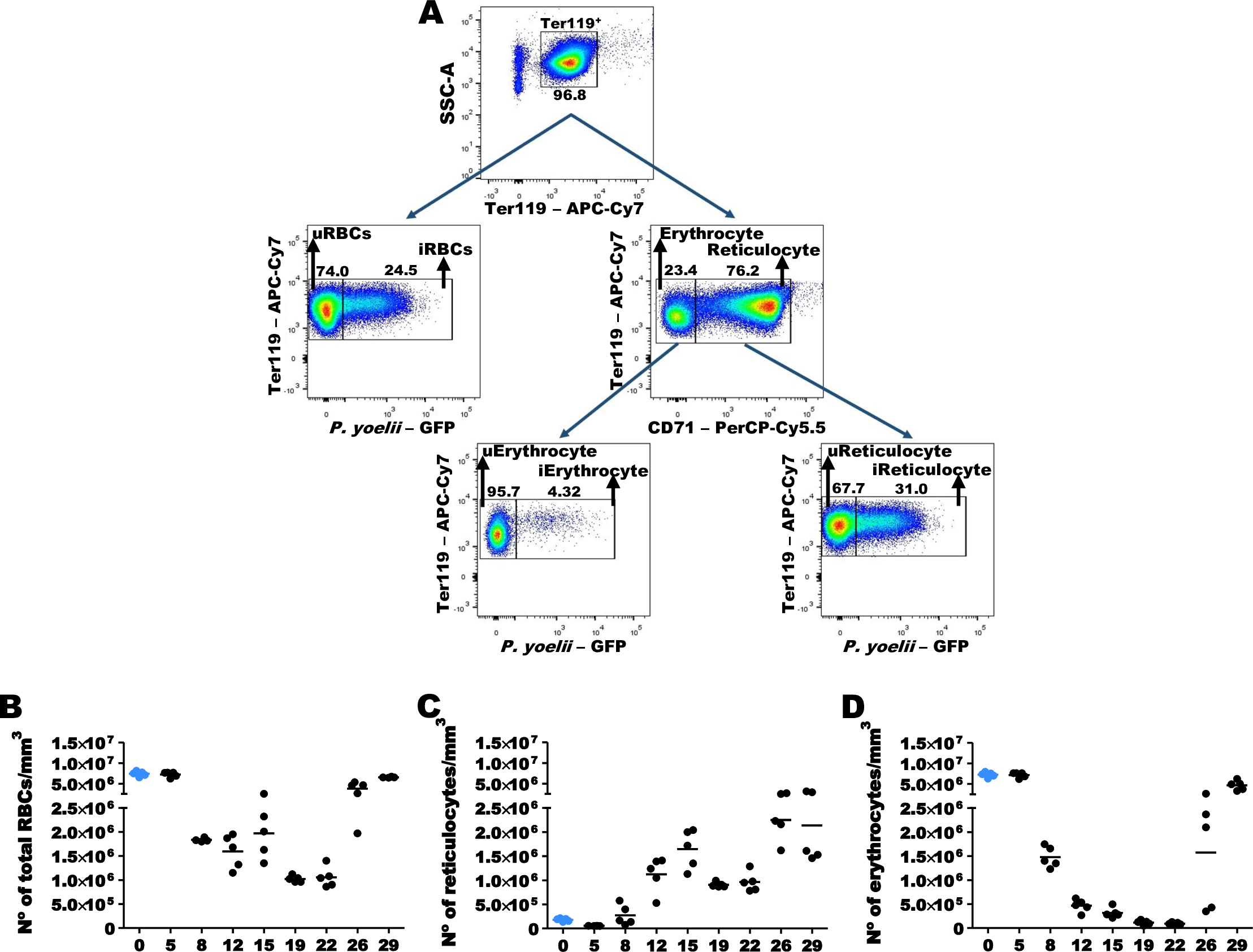

Supplement: S1 Fig — (A) Gating strategy for the analysis of parasitemia. RBCs count during P. yoelii infection, presented as total RBCs (B), Retics (C) and erythrocytes (D). (TIF) [file ppat.1008840.s001.tif]

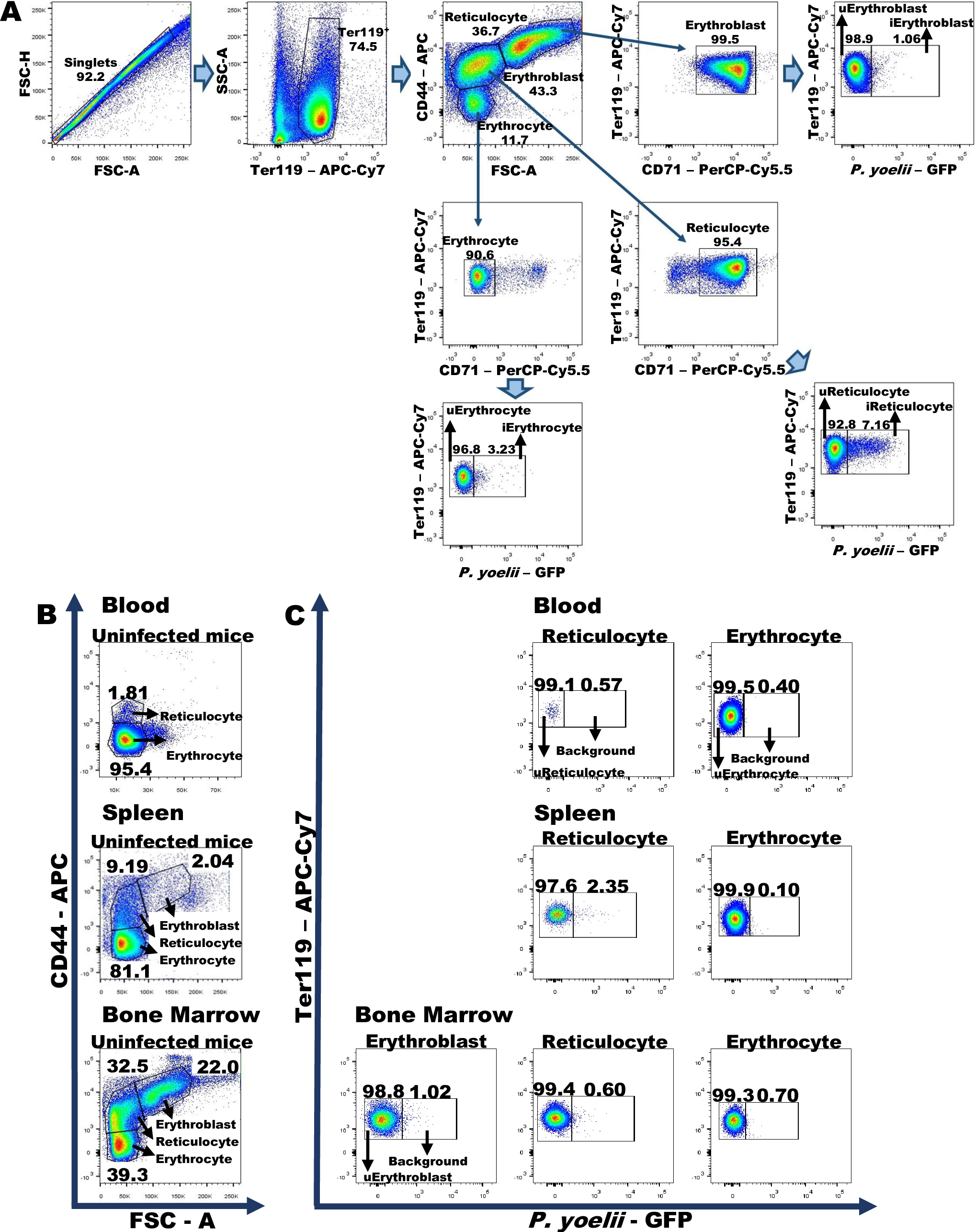

Supplement: S2 Fig — (A) Gating strategy for the analysis of RBC subsets and GFP+ P. yoelii-infected cells within these subsets. (B) Representative dot-plots show the frequency of erythroblasts, Retics and erythrocytes in blood, spleen and bone marrow from uninfected mice. (C) Evaluation of GFP+ control background in RBCs subsets from uninfected mice. (TIF) [file ppat.1008840.s002.tif]

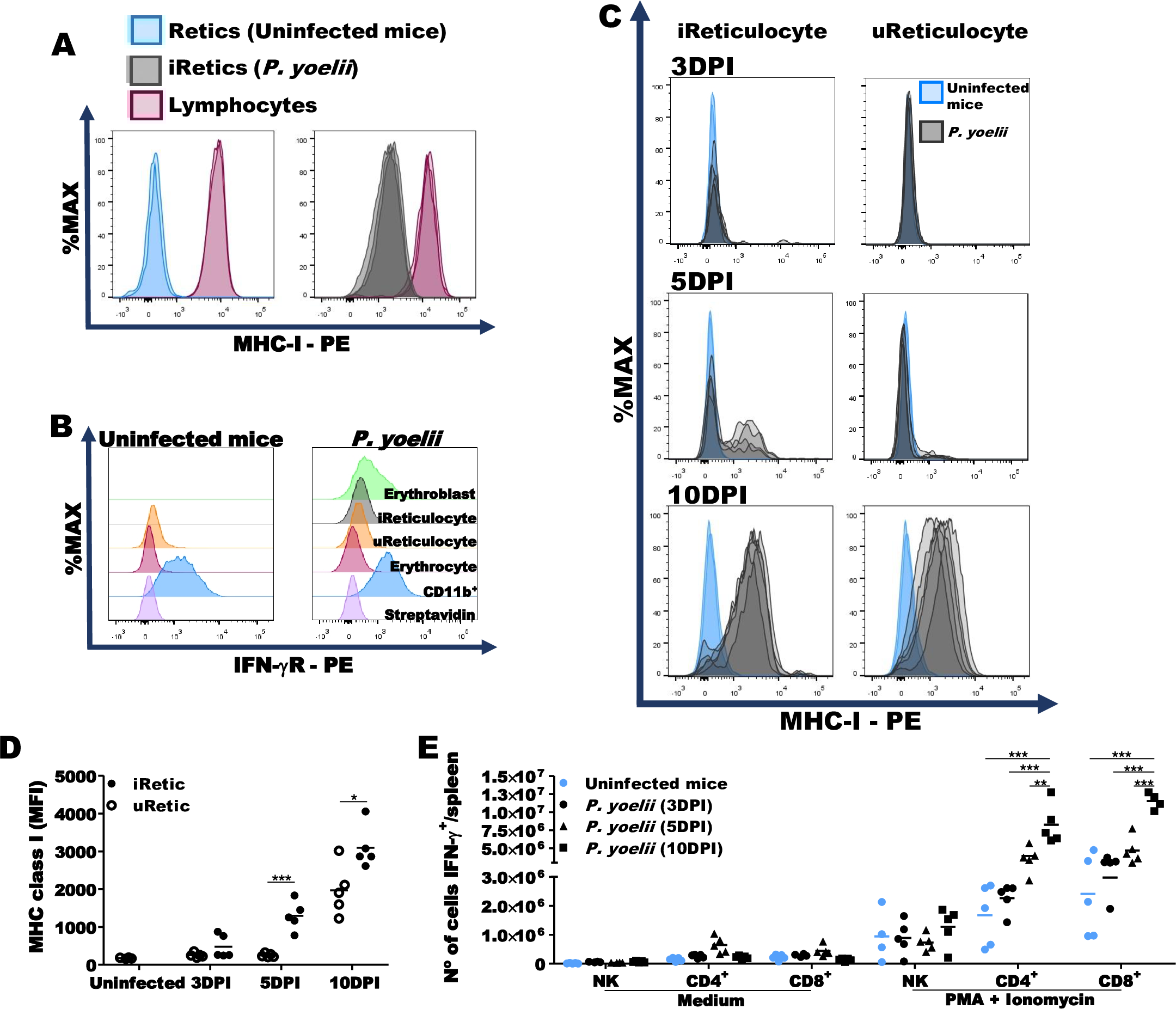

Supplement: S3 Fig — (A) The expression of MHC-I on Retics and lymphocytes (magenta) was compared between uninfected mice (blue) and P. yoelii-infected mice (grey). iRetics showed higher levels of MHC-I compared to Retics from uninfected mice. However, iRetics express about a log less MHC-I than splenic lymphocytes. Similar levels of MHC-I expression were observed in lymphocytes regardless of infection. (B) Representative histograms of IFN-γR in RBCs subsets and CD11b+ from uninfected and P. yoelii-infected mice at 12 DPI. Purple histogram represent background (sample stained with streptavidin only). (C) Representative histograms of MHC-I expression on iRetics and uRetics from blood of infected mice at 3, 5 and 10DPI. (D) iRetics (filled circles) express MHC-I on their surface earlier than uRetics (open circles) in P. yoelii-infected mice. (E) The number of IFN-γ-producing T cells increases during the course of infection and concur with emergence of MHC-I on Retics surface. (TIF) [file ppat.1008840.s003.tif]

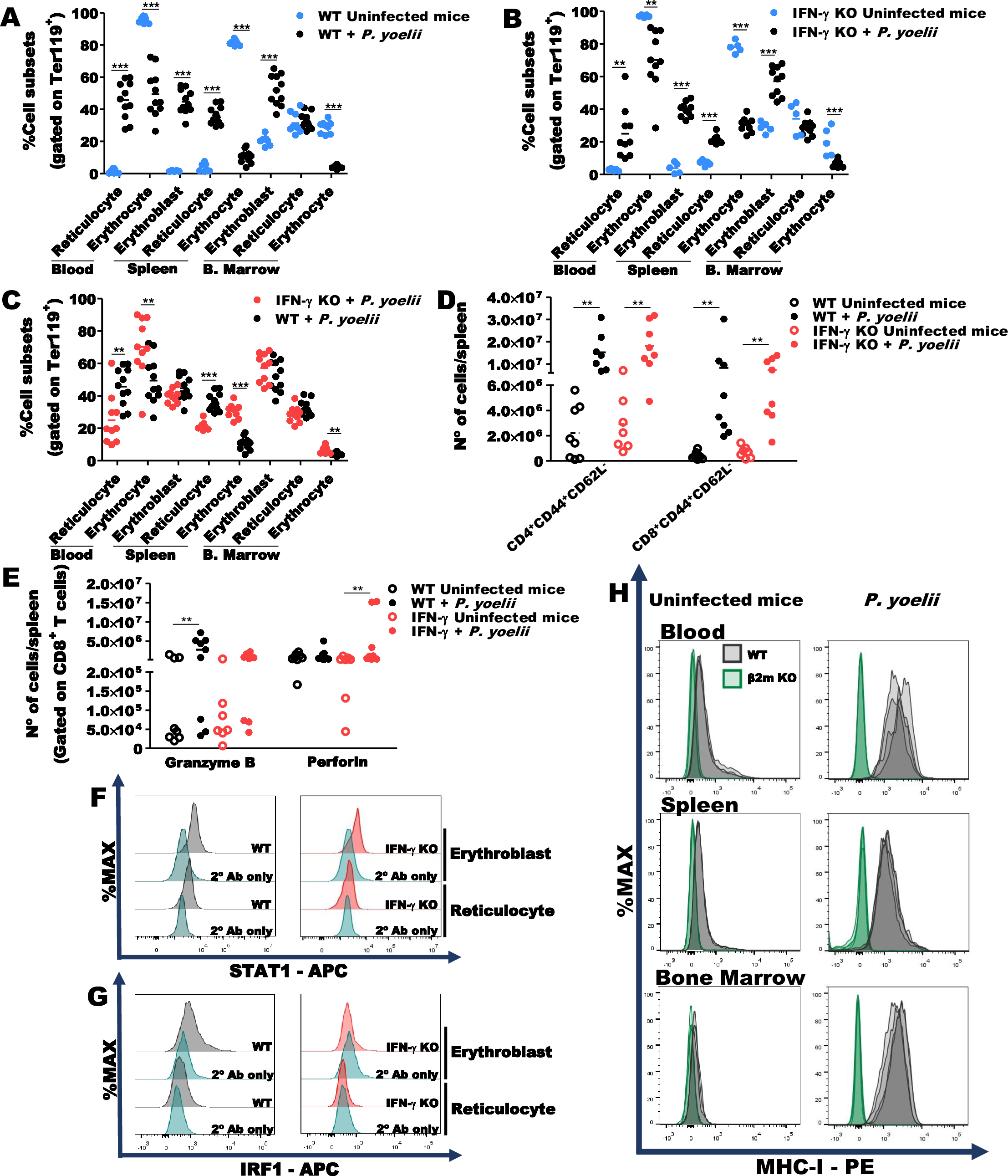

Supplement: S4 Fig — P. yoelii-infected mice show an increase in the percentage of reticulocytes in the blood and spleen both in WT (A) and IFN-γ KO (B) mice. However, reticulocytosis is less pronounced in IFN-γ KO infected mice (C). (D) The number of CD4+ and CD8+ T cells expressing activation markers (CD44+ and CD62L-) during P. yoelii infection is increased both in WT (black circles) and IFN-γ KO (red circles) mice. (E) P. yoelii-infected IFN-γ KO mice (filled red circles) do not increase the number of CD8+ T cells producing granzyme B unlike infected WT mice (filled black circles). However, the number of CD8+ T cells producing perforin is increased in IFN-γ KO mice (filled red circles) 12 DPI. Representative dot-plots of STAT1 (F) and IRF1 (G) levels in P. yoelii-infected WT (grey) and IFN-γ KO mice (red) at 12 DPI. The green histograms represent background (samples stained with secondary antibody only). (H) β2-m KO mice (green) do not express MHC-I. Representative histograms of Retics from blood, spleen and bone marrow. Retics and lymphocytes were isolated from uninfected mice or P. yoelii-infected mice at 12 DPI. The statistical analysis of RBCs subsets was performed using unpaired t-test or Mann-Whitney U test, according to the normality of data distribution. Data are pooled from two-three independent experiments (WT: n = 9, uninfected mice; n = 11, P. yoelii-infected mice; IFN-γ KO: n = 5, uninfected mice; n = 10, P. yoelii-infected mice). The statistical analysis of T cells was performed using unpaired t-test or Mann-Whitney U test, according to data distribution. Data are pooled from two independent experiments (n = 8 for each groups). **p<0.01; ***p<0.001. (TIF) [file ppat.1008840.s004.tif]

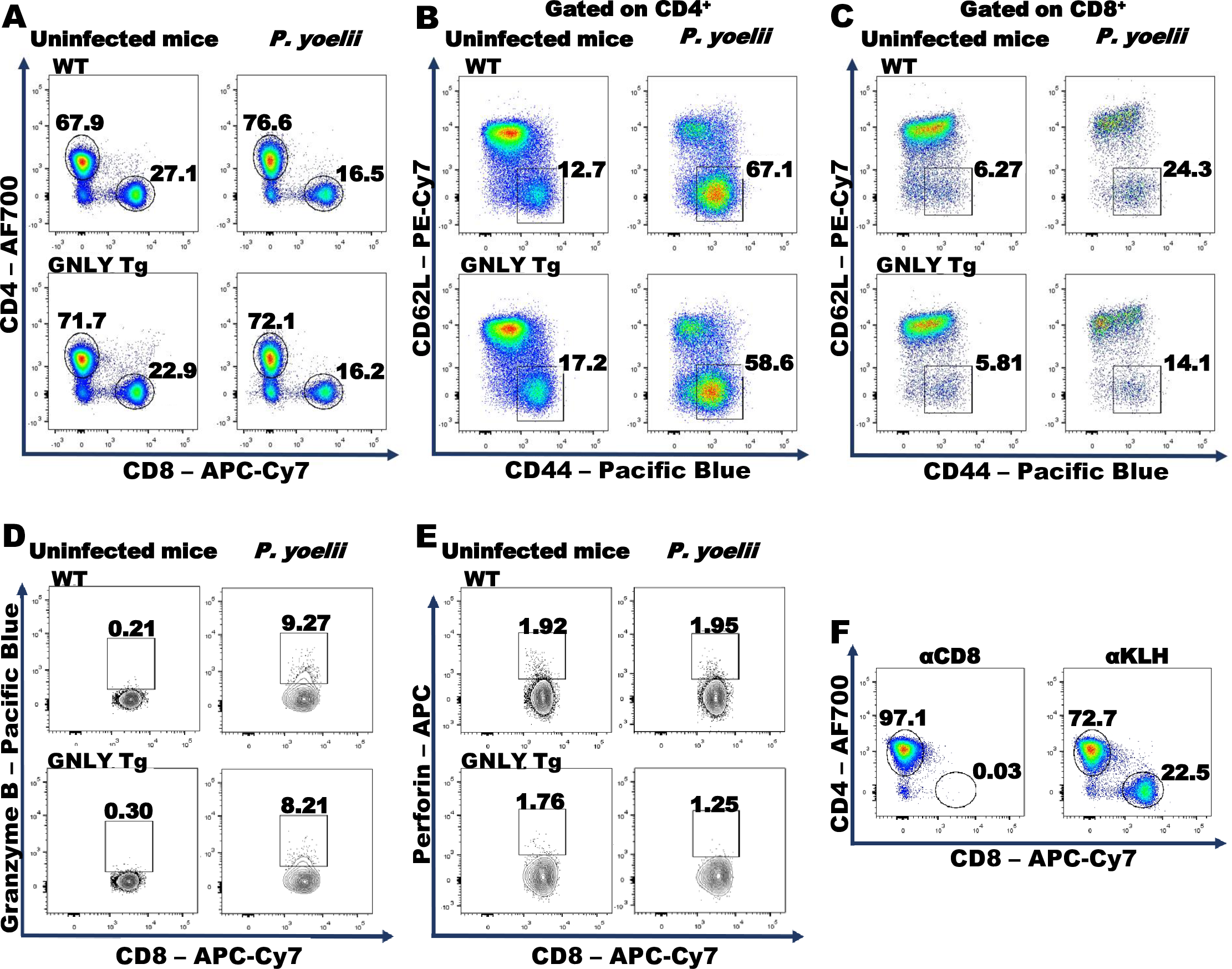

Supplement: S5 Fig — (A) Dot plots of CD4+ and CD8+ T cell subsets from WT (upper panels) and GNLY-Tg (lower panels). Activation status dot plots of CD4+ T (B) and CD8+ T (C) cells from WT (upper panels) and GNLY-Tg (lower panels). Granzyme B (D) and Perforin (E) dot plots of splenic CD8+ T cells in WT (upper panels) and GNLY-Tg (lower panels). Analyses were performed using splenocytes from uninfected or P. yoelii-infected mice 12 DPI. (F) Dot plot of CD4+ and CD8+ T cells subsets from GNLY-Tg mice depleted of CD8+ T cells (left panel) and GNLY-Tg mice treated with αKLH (right panel). This analysis was performed 3 days post antibody treatment. (TIF) [file ppat.1008840.s005.tif]
